# A Rare Grotesque Skeletal Deformity: Munchmeyer’s Disease

**DOI:** 10.31138/mjr.32.1.91

**Published:** 2021-03-31

**Authors:** Debashish Mishra, Aadhaar Dhooria

**Affiliations:** 1 Department of Internal Medicine, Rheumatology and Clinical Immunology, Post Graduate Institute of Medical Education and Research, Chandigarh, India; 2 Consultant Rheumatologist, Department of Rheumatology, Santokba Durlabhji Memorial Hospital, Jaipur, India

**Keywords:** Fibrodysplasia, myositis, ossificans, genetic, musculoskeletal

Fibrodysplasia ossificans progressiva (FOP), or Munchmeyer’s disease, is a rare deforming skeletal anomaly caused by intramuscular ossification. Here, we present the clinical images of a patient with this rare grotesque deforming disease.

**Figure 1.**
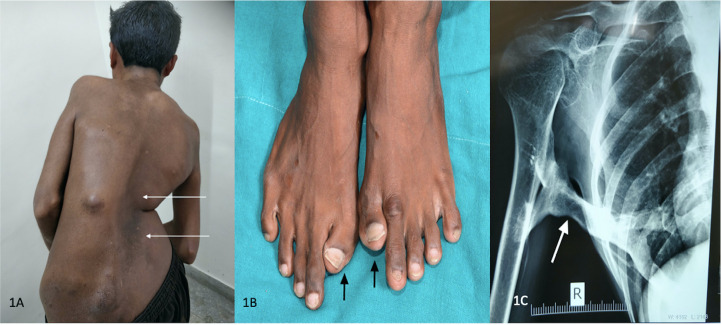
Clinical photograph showing kyphoscoliosis (**A**) and short first toe in both feet (**B**) and radiograph showing ossification of right latissimus dorsi muscle (**C**).

A 20-year-old gentleman presented to our clinic with inability to open his mouth and difficulty in eating. Progressive deformities had been noticed starting at the age of two and a half months, following intramuscular tetanus immunisation. At present, patient was unable to abduct his arms or stand straight. Examination revealed a hard sheet-like structure binding his arms to the chest and kyphoscoliosis (**Figure 1A**) and shortened great toes (**Figure 1B**). Hip movements were limited. A hard submandibular swelling was noted, grossly limiting jaw movements. Radiographs revealed ossification of latissimus dorsi bilaterally (**Figure 1C**) and monophalangic great toes, confirming a diagnosis of FOP. Patient and family was counselled about the disease, aggravating factors, and avoidance of intramuscular injections.

FOP is a rare disorder (1 in 2,000,000), caused by an autosomal dominant mutation in ACVR1 gene on chromosome 2q 23–24, encoding bone morphogenetic protein (BMP) type1 receptor.^1^ There is no proven treatment yet, except for several trials with nitrogenous bisphosphonates,^2^ and few ongoing ones with drugs like palovarotene (MOVE), garetosmab, (LUMINA-1), rapamycin, and saracatinib (STOPFOP group). Avoidance of any sort of trauma is primary to prevent disease progression.
